# Beyond Hemolysis: Babesiosis-Associated Atraumatic Splenic Infarction in an Immunocompetent Patient

**DOI:** 10.7759/cureus.105838

**Published:** 2026-03-25

**Authors:** Jenny Joseph, Brandon Bharat, Peter Huh

**Affiliations:** 1 Department of Medicine, Norwalk Hospital, Norwalk, USA; 2 Department of Pulmonology and Critical Care Medicine, Norwalk Hospital, Norwalk, USA

**Keywords:** babesia microti, babesiosis, hemolytic anemia, parasite infection, parasitemia index, peripheral blood smear, splenic infarction, tick-borne infections

## Abstract

Babesiosis is a protozoan infection transmitted by the tick, *Ixodes scapularis*. The disease presentation varies from asymptomatic to fatal illness. Usually, severe illness occurs in asplenic and immunocompromised patients. In rare cases, the complication of splenic injury, including infarcts and rupture, has been observed in young individuals. We present the case of a middle-aged woman who presented with abdominal pain and fatigue. She was found to be febrile and hypotensive with thrombocytopenia, hemolytic anemia, and elevated transaminases. Imaging of the abdomen demonstrated multiple splenic infarcts. The patient denied a history of abdominal trauma and tick exposure. Autoimmune and infective endocarditis workup was negative. Subsequently, she tested positive for *Babesia microti* by polymerase chain reaction (PCR) test, and peripheral smear was remarkable for intracellular and extracellular ring forms consistent with *Babesia* with a parasitemia index of 1%. She tested negative for Lyme disease and anaplasmosis by enzyme-linked immunosorbent assay (ELISA) and PCR, respectively. She was started on atovaquone and azithromycin. On day 8 of hospitalization, she was transfused with one unit of packed red blood cells due to a hemoglobin level of 6.8 g/dL. Repeat parasitemia index on discharge was 0.01%. She was later discharged without further need for blood transfusions or surgical interventions. This case highlights the importance of considering babesiosis as a cause of acute splenic infarcts in endemic regions, even without reported exposure.

## Introduction

Babesiosis is a zoonotic infection caused by intraerythrocytic protozoa of the genus *Babesia*, most commonly *Babesia microti* in the United States. Transmission most commonly occurs through bites from the *Ixodes scapularis* tick, although cases related to blood transfusion have also been reported [1]. Vertical transmission has additionally been described, highlighting alternative mechanisms of disease spread outside of tick exposure [2]. Once introduced into a human host, *Babesia* organisms invade erythrocytes and replicate intracellularly, leading to hemolysis and a systemic inflammatory response. Studies have demonstrated that elevated inflammatory cytokines, including tumor necrosis factor-α and interleukin-6, regulate the immune response during acute infection and contribute to the clinical manifestations of infection [3]. The incidence of babesiosis has increased significantly over the past two decades, particularly in the northeastern and upper Midwestern regions of the United States, where *Ixodes scapularis* ticks are endemic [4]. Clinical manifestations vary and may range from asymptomatic infections to severe illness characterized by fever, fatigue, hemolytic anemia, thrombocytopenia, and elevated liver enzymes [5]. Severe disease is more frequently observed in older adults, immunocompromised individuals, and patients with functional or anatomical asplenia [6]. Although hemolysis represents the hallmark manifestation of babesiosis, rare complications involving the spleen, such as splenic infarction or rupture, have been reported [7-9]. These complications can occur disproportionately in younger immunocompetent individuals with relatively low levels of parasitemia [9]. We present a case of splenic infarction secondary to babesiosis in an immunocompetent patient without reported tick exposure.

## Case presentation

**Figure 1:**
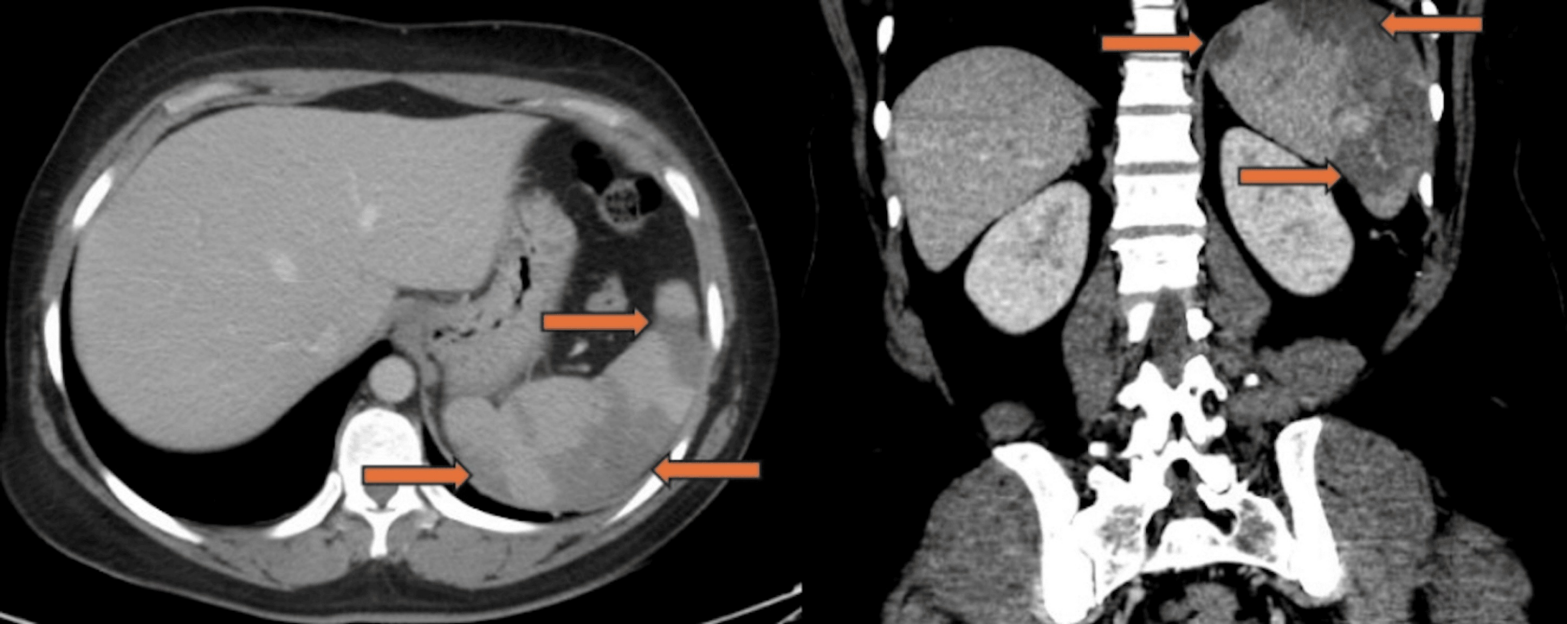
Splenic infarctions on contrast-enhanced CT abdomen in disseminated babesiosis. Axial (left) and coronal (right) contrast-enhanced CT images of the abdomen demonstrating multiple peripheral wedge-shaped hypoattenuating regions within the spleen (orange arrows), consistent with splenic infarctions.

**Figure 2:**
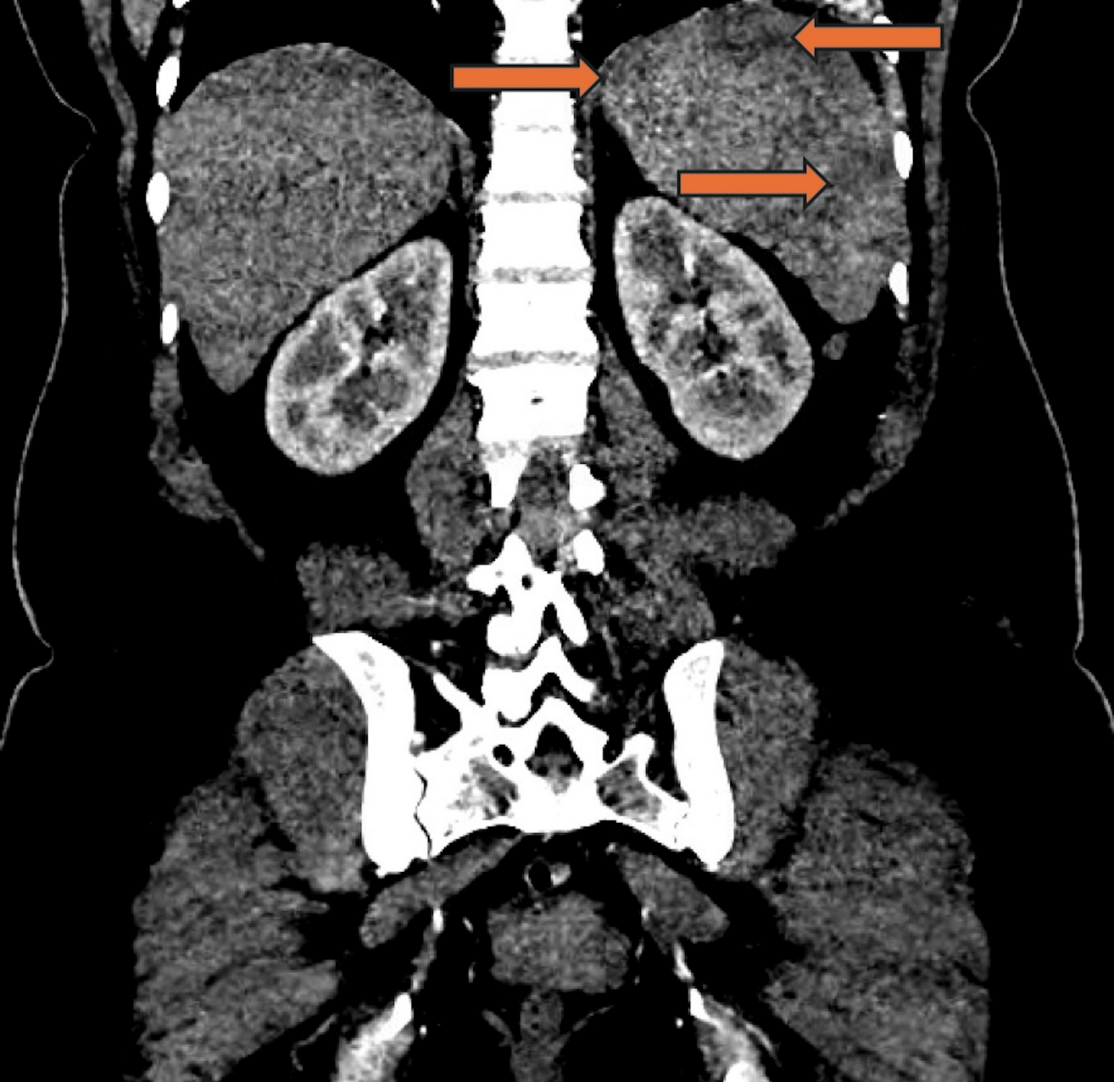
Splenic infarctions on CT angiography (CTA) in disseminated babesiosis. Coronal contrast-enhanced CTA of the abdomen demonstrating multiple peripheral wedge-shaped hypoattenuating areas within the spleen (orange arrows), consistent with splenic infarctions.

**Figure 3:**
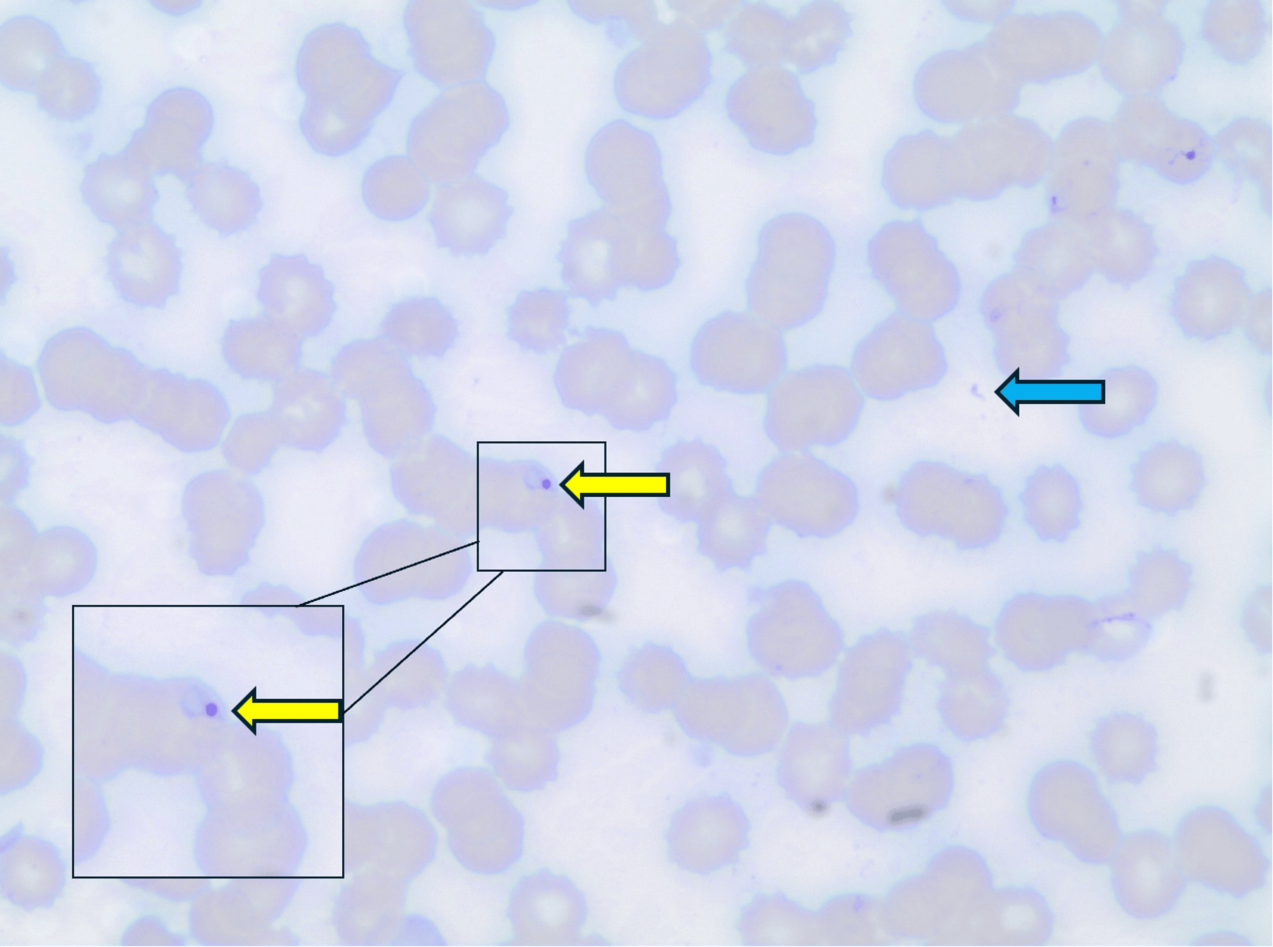
Peripheral smear showing intracellular and extracellular ring formations consistent with Babesia infection. Giemsa-stained peripheral blood smear demonstrating an intraerythrocytic ring-form parasite consistent with a Babesia trophozoite (yellow arrows), with magnified inset for clarity. An extracellular parasite consistent with a free merozoite is also seen (blue arrow), likely representative of release following erythrocyte rupture.

A 48-year-old female with no past medical history or trauma presented to the hospital in the northeastern United States with abdominal pain. On admission, she was febrile, hypotensive, and saturating well on room air. Pertinent physical examination findings included diffuse abdominal tenderness. Initial laboratory studies demonstrated thrombocytopenia, hemolytic anemia with elevated lactate dehydrogenase and decreased haptoglobin, and elevated transaminases (Table 1). An electrocardiogram was notable for a normal sinus rhythm. She responded appropriately to initial fluid resuscitation and was hemodynamically stable. Computed tomography (CT) scan of abdomen and pelvis with contrast demonstrated multiple splenic infarcts (Figure 1), which were redemonstrated on CT angiography of the abdomen and pelvis (Figure 2). CT angiography of the abdomen and pelvis also ruled out abnormalities of the vasculature. Autoimmune workup was grossly unremarkable and only notable for antinuclear antibody (ANA) of 1:320. The positive ANA was considered to be nonspecific and not clinically consistent with autoimmune disease in the absence of other supporting findings. A transthoracic echocardiogram (TTE) showed normal ejection fraction without any regional wall motion abnormalities, ruling out Lyme carditis. She was also noted to have a downtrending C-reactive protein (CRP) from 202 mg/L on admission, along with improvement of liver enzymes. Subsequently, she tested positive for *Babesia microti* using polymerase chain reaction (PCR). *Anaplasma* and *Ehrlichia* PCR tests were negative. Although she tested positive on the Lyme total antibody screening assay, her confirmatory IgG and IgM levels were negative with enzyme-linked immunosorbent assay (ELISA). Hence, she began treatment with oral atovaquone and azithromycin for babesiosis. Peripheral smear was remarkable for intracellular ring forms and extracellular parasites (Figure 3), consistent with *Babesia* and parasitemia of approximately 1% of erythrocytes. Repeat parasitemia index on discharge was 0.01%. She was then discharged with atovaquone and azithromycin to complete a 10-day course.

**Table 1:** Initial laboratory evaluation demonstrating thrombocytopenia, hemolytic anemia, elevated transaminases, and inflammatory marker elevation. Diagnostic testing confirmed Babesia microti infection by polymerase chain reaction with peripheral smear demonstrating intraerythrocytic rings. PCR: polymerase chain reaction

Laboratory test	Result	Reference range
Hemoglobin	10.7 g/dL	12-16 g/dL
Lactate dehydrogenase (LDH)	977 U/L	122-222 U/L
Haptoglobin	<10 mg/dL	30-200 mg/dL
Total bilirubin	1.9 mg/dL	0.0-1.2 mg/dL
Indirect bilirubin	0.9 mg/dL	0.0-0.3 mg/dL
Reticulocyte count	3 %	0.5-2.5 %
White blood cells	5.7 × 10^9^/L	3.5-10 × 10^9^/L
Bands	6 %	0-4 %
Platelets	83 × 10^9^/L	150-400 × 10^9^/L
Alkaline phosphatase	410 U/L	30-103 U/L
Alanine aminotransferase (ALT)	199 U/L	10-55 U/L
Aspartate aminotransferase (AST)	243 U/L	10-50 U/L
CRP	202.7 mg/L	0.0-4.9 mg/L
ANA titer	1:320	<1:80
*Babesia* PCR	Detected	Detected/not detected
*Anaplasma* PCR	Not detected	Detected/not detected
*Ehrlichia* PCR	Not detected	Detected/not detected
Lyme total antibody screen	Positive	Positive/negative
Lyme confirmatory IgG/IgM	Negative	Positive/negative
Peripheral smear	Positive for intracellular and extracellular ring forms	None detected
Parasitemia index on admission	1%	None detected
Parasitemia index on day 7	0.01%	None detected
Blood culture	No growth	No growth

## Discussion

Although *Babesia* is commonly transmitted by *Ixodes scapularis* tick bites, it can also be transmitted through transfusion of blood products [1] and solid organ transplants from asymptomatic donors, and transplacental transmission [2]. Since *Ixodes scapularis* ticks are also reservoirs for other pathogens like *Borrelia*, *Anaplasma*, and *Ehrlichia*, co-infection with Lyme disease, anaplasmosis, and ehrlichiosis is common in babesiosis. After entry into human hosts, the *Babesia* spp. invade erythrocytes and begin the intraerythrocytic cycle during which the merozoites are released from the red blood cells, causing hemolysis and subsequent anemia. The spleen plays a critical role during active infection as macrophages aid in the clearance of infected erythrocytes. Infection also results in a cytokine storm with tumor necrosis factor-alpha and interleukin-6 [3], which leads to symptoms including fever, headache, myalgia, and, in some cases, acute lung injury.

Over the last three decades, the incidence of babesiosis has significantly increased in the northeastern region of the United States [4]. The clinical presentation varies from asymptomatic to fatal illness. About 20% of adults with babesiosis are asymptomatic [5]. The most common symptoms include chills, myalgia, headache, nausea, and anorexia. Severe illnesses, including massive hemolytic anemia, disseminated intravascular coagulation, and multi-organ failure, are usually seen in asplenic, immunocompromised, and elderly patients [6]. There are a few reported cases of splenic infarction as a complication of babesiosis [7-9]. A review of literature revealed just 35 documented cases of *Babesia*-associated splenic injury through 2021 [8]. Similar to previously reported cases, splenic infarction in babesiosis appears to occur disproportionately in younger, immunocompetent patients with relatively low levels of parasitemia [7,9].

The pathophysiology of splenic injury related to babesiosis is not well understood. One of the suggested mechanisms is like that seen in malaria, which includes the inability of the affected erythrocytes to deform and their increased adhesion to the capillary walls, thus causing vascular obstruction [9]. Froberg et al. reported a case of splenic rupture caused by babesiosis, and the splenic biopsy samples noted erythrophagocytosis [10]. Besides rapid sequestration of the infected RBC and platelets by the spleen, many of these RBCs also demonstrated immunoreactivity against *Babesia* antibodies. A combination of these factors could lead to splenic enlargement and eventual rupture. As in our patient, most of the reported cases of babesiosis-induced splenic injury are in young, immunocompetent patients with low levels of parasitemia. This paradox can be explained by the fact that younger, healthier patients have a robust defense mechanism, which results in increased mechanical strain on the spleen [11].

Treatment for mild to moderate babesiosis typically consists of oral azithromycin dosed at 500 mg once daily on day 1, followed by 250 mg once daily beginning on day 2 and atovaquone 750 mg every 12 hours for a total duration of seven to 10 days in both immunocompetent and immunocompromised patients [12]. In cases of severe babesiosis, which is associated with parasitemia greater than 4%, intravenous azithromycin is preferred over oral therapy. Clindamycin plus quinine is an alternative, but tolerability is poor. Our patient improved with oral therapy despite splenic infarction, which could be explained by low levels of parasitemia. Exchange transfusion must be considered in patients with parasitemia greater than 10% or severe hemolytic anemia or end-organ damage.

## Conclusions

Most cases of babesiosis-induced splenic infarction are managed conservatively with antimicrobial therapy. Splenectomy must be reserved for patients with hemorrhagic shock. It is important to consider babesiosis as a cause of splenic infarction even in young immunocompetent adults, as failure to treat underlying *Babesia* and inadvertent splenectomy can result in massive hemolysis. This case also highlights that it is important to consider babesiosis as a cause of splenic infarction even in young immunocompetent adults, as an intact immune system promotes efficient clearance of infected erythrocytes, which may place significant mechanical strain on the spleen.
